# Mechanisms Underlying the Beneficial Effects of Scraping Therapy: A Review of Articles

**DOI:** 10.7759/cureus.114412

**Published:** 2026-08-12

**Authors:** Yi Dan Chu, Eric Chun-Pu Chu

**Affiliations:** 1 Chiropractic and Physiotherapy Centre, New York Medical Group, Hong Kong, CHN

**Keywords:** gua sha, immune modulation, instrument-assisted soft tissue mobilization petechiae, microhemolysis, petechiae, physiological mechanisms, scraping therapy

## Abstract

Scraping therapy, known as Gua Sha in traditional Chinese medicine, spoon-rubbing in folk therapy, or instrument-assisted soft tissue mobilization in contemporary physical therapy, entails applying controlled scraping pressure to the skin to deliberately induce petechiae and ecchymosis. This mechanical stimulus is believed to trigger a subsequent biological response that may reduce inflammation and alleviate pain. It is frequently employed to address neck pain, shoulder tension, muscle soreness, chronic pain, and related muscle problems. With the growing burden of noncommunicable diseases among aging populations, there is increasing focus on distributing healthcare responsibilities to non-physician practitioners. Nevertheless, the precise physiological mechanisms underlying its clinical benefits remain incompletely understood and warrant further scientific validation. This presentation seeks to reinterpret this traditional therapeutic approach by integrating it with current scientific knowledge and modern medical frameworks.

## Introduction and background

Scraping therapy (Gua Sha) is a manual therapeutic practice that originated over 2000 years ago in China and Southeast Asia [1]. In traditional terminology, the word "Gua" means to scrape or stroke, while "Sha" carries a dual definition: it denotes both the underlying physiological stasis of blood and fluids and the resulting transient petechiae and ecchymosis elicited on the skin surface. Gua Sha involves repeatedly scraping the skin with a smooth-edged tool until minor bruising or petechiae occur [1]. This therapy aims to reduce microenvironmental stagnation and improve circulation in the treated area. The tools used vary according to the therapeutic regimen; for example, a porcelain spoon or a buffalo horn scraper is commonly used, polished stainless steel is useful for breaking up scar tissue, and heated tools are normal for reducing muscle strain. It is widely used in contemporary clinical practice to alleviate muscle tension, aid in athletic recovery, and modulate immune responses to reduce inflammation [2,3]. Scientists are actively investigating the mechanisms behind its observed clinical benefits, driving substantial research interest in exploring its physiological effects through rigorous biomedical studies [4,5]. Specifically, empirical investigations have demonstrated that scraping therapy induces microvascular hyperemia to enhance local perfusion, upregulates heme oxygenase-1 (HO-1) to exert systemic anti-inflammatory and antioxidant effects, modulates peripheral and central pain signaling pathways, and influences autonomic nervous system tone. Clinically, these physiological responses have been evaluated in conditions ranging from chronic neck and low back pain to diabetic peripheral neuropathy and musculoskeletal fatigue [4,5].

In clinical and biological terms, scraping therapy involves mechanical friction that induces petechial markings, which later resolve through microhemolysis [6-9]. Research suggests the purported effectiveness results from controlled microvascular injury triggering cytoprotective enzymes, systemic immune-modulating signals, and neural mechanisms like pain modulation, endogenous opioid release, and autonomic recalibration [1,6-9]. This paper explores each of these mechanistic domains, integrating findings from animal studies, translational research, and clinical data to offer an up-to-date explanation of how scraping therapy may produce its therapeutic effects.

## Review

Microvascular disruption and the hemolytic cascade

Scraping therapy works by gently breaking tiny blood vessels near the skin's surface. This controlled extravasation appears to start the body's healing process [1]. Laser Doppler imaging shows significant increases in microperfusion and a localized rise in body surface temperature (up to 1°C) in the scraped area [2]. The improved perfusion allows for a more rapid exchange of oxygen, nutrients, and immune cells within the interstitial fluid [10-12]. As extravasated erythrocytes are reabsorbed, they undergo autologous hemolysis, which triggers the upregulation of HO-1 gene expression [13]. HO-1 is a stress-inducible enzyme that catalyzes the degradation of free pro-oxidant heme into biliverdin, carbon monoxide (CO), and ferrous iron [14,15]. Biliverdin acts as an antioxidant, while CO stimulates interleukin-10 (IL-10, an anti-inflammatory cytokine) to suppress the production of inflammatory mediators such as IL-1β, IL-6, IL-8, and TNF-α. Simultaneously, the oxidation of released ferrous iron further reduces the local oxidative burden [16]. Tissue breakdown also remodels the local capillary network and stimulates lymphatic drainage, which enhances the speed of substance exchange among blood, interstitial fluid, and lymphatic fluid [12,13].

HO-1 as a central cytoprotective mediator

The strongest evidence linking scraping therapy to reduced inflammation involves the long-lasting increase in an enzyme called HO-1. This enzyme responds strongly to hypoxia and stress and has potent antioxidant, anti-inflammatory, and immunomodulatory properties in vascular cells [13-15]. Researchers at Harvard Medical School used bioluminescent imaging in mice to show that just one scraping session boosts HO-1 levels for a prolonged time [1]. This protective response is triggered by the release of circulating hemoglobin debris following capillary rupture [1]. The HO pathway in the spinal cord further modulates nociception originating in peripheral tissues, which links the process of erythrocyte breakdown to pain relief [4]. Together, these effects show how activating one enzyme can turn a simple mechanical stimulation into systemic anti-inflammatory benefits.

Immune modulation: cytokine recalibration and immune cell activation

In addition to inducing HO-1, scraping therapy produces direct and quantifiable impacts on immune cell profiles and cytokine dynamics, both locally and systemically. It provides lasting pain relief, improved mobility, and longer-lasting reductions in TNF-α (a biomarker of inflammation) compared to hot compress therapy, as shown in a pilot study of older adults with chronic back pain [17]. Moreover, decreased TNF-α levels after scraping therapy were closely associated with reduced physical disability. Research in animals suggests that scraping therapy boosts immune alertness by modulating cytokines locally, distantly, and systemically [5]. In another animal study on herniated disc, scraping therapy reduced serum pro-inflammatory cytokines (IL-1β, IL-6, and TNF-α) and reduced pain sensitivity to heat, demonstrating that its pain-relieving benefits are closely tied to underlying immunomodulatory mechanisms [11].

Tu et al. found that scraping therapy combined with bloodletting reduced systemic inflammation and organ damage in heatstroke-model rats [18]. A 2025 study on radiculitis rats further demonstrated that scraping downregulates inflammatory markers (IL-1β, TNF-α, NF-κB) and modulates HIF-1α-mediated glycolysis, suggesting a localized metabolic effect on sensitized nerves [6]. Overall, scraping therapy enhances immune surveillance by boosting both innate and adaptive skin immunity while also tempering excessive and destructive inflammation by the suppression of specific pro-inflammatory and immunosuppressive cytokines [5].

Neurological mechanisms of analgesia

Scraping therapy activates multiple neurological pathways to relieve pain, with these pathways overlapping rather than competing. The gate control theory of pain suggests that a "gate" in the spinal cord controls pain signal transmission to the brain. Naturopathic practices like massage, Gua Sha, and cupping stimulate mechanoreceptors that compete with pain signals in the spinal cord, modulating pain before it reaches the brain [19]. Additionally, manual therapies can induce a "counter-irritation" response, a physiological process where irritation at one site influences pain perception elsewhere, thereby extending pain relief beyond the immediate treatment area [20].

A second pathway involves the release of endogenous bioactive substances. Endothelial cell production of nitric oxide (NO) is stimulated by shear stress from scraping. The synthesized NO relaxes vascular smooth muscle, improves tissue oxygenation, suppresses inflammatory pain signals, and reduces edema [13,15,16]. Evidence from 75 studies supports manual therapy's role in modulating endogenous opioid systems and potential use in addiction treatment [9]. A rat model of chronic soft tissue injury showed that scraping increased β-endorphin levels in immune tissue, decreased inflammatory markers, and reduced M1 macrophage response [21]. This represents the in vivo evidence that scraping induces rises in tissue β-endorphin levels, coinciding with both anti-inflammatory changes and pain-relieving effects. An analogous finding is provided by Kaada and Torsteinbø, who assessed plasma β-endorphins in 12 volunteers before and after a 30-minute connective tissue massage, observing a 16% increase in plasma β-endorphins that persisted for over an hour [22]. It is important to note, however, that direct human clinical trials quantifying plasma β-endorphin dynamic changes specifically following Gua Sha or scraping therapy remain absent from the current literature. Because of this gap in human clinical data, understanding of scraping-induced endogenous opioid pathways currently relies on preclinical animal models [21] and translational proxies from related human manual therapies [22]. Further direct human trials measuring post-scraping circulating endorphins are necessary to confirm these neurohumoral mechanisms in clinical populations. While this intervention differs from scraping therapy, it indicates that intense mechanical skin stimulation can raise circulating β-endorphins, supporting the neurobiological basis for the pain relief provided by scraping therapy.

Autonomic modulation and psychological responses

Scraping therapy produces psychological benefits such as lower heart rate, reduced cortisol levels, and emotional calm through measurable autonomic and neurochemical changes, including the activation of the parasympathetic system and decreased sympathetic activity. Heart rate variability (HRV) serves as a key indicator of these effects. After scraping therapy, time-domain HRV metrics typically increase, reflecting a shift toward enhanced parasympathetic (vagal) nerve activity and improved autonomic balance [23]. It is plausible that rhythmic pressure stimulating skin receptors signals the brainstem to shift autonomic balance toward parasympathetic dominance [24-26].

**Figure 1:**
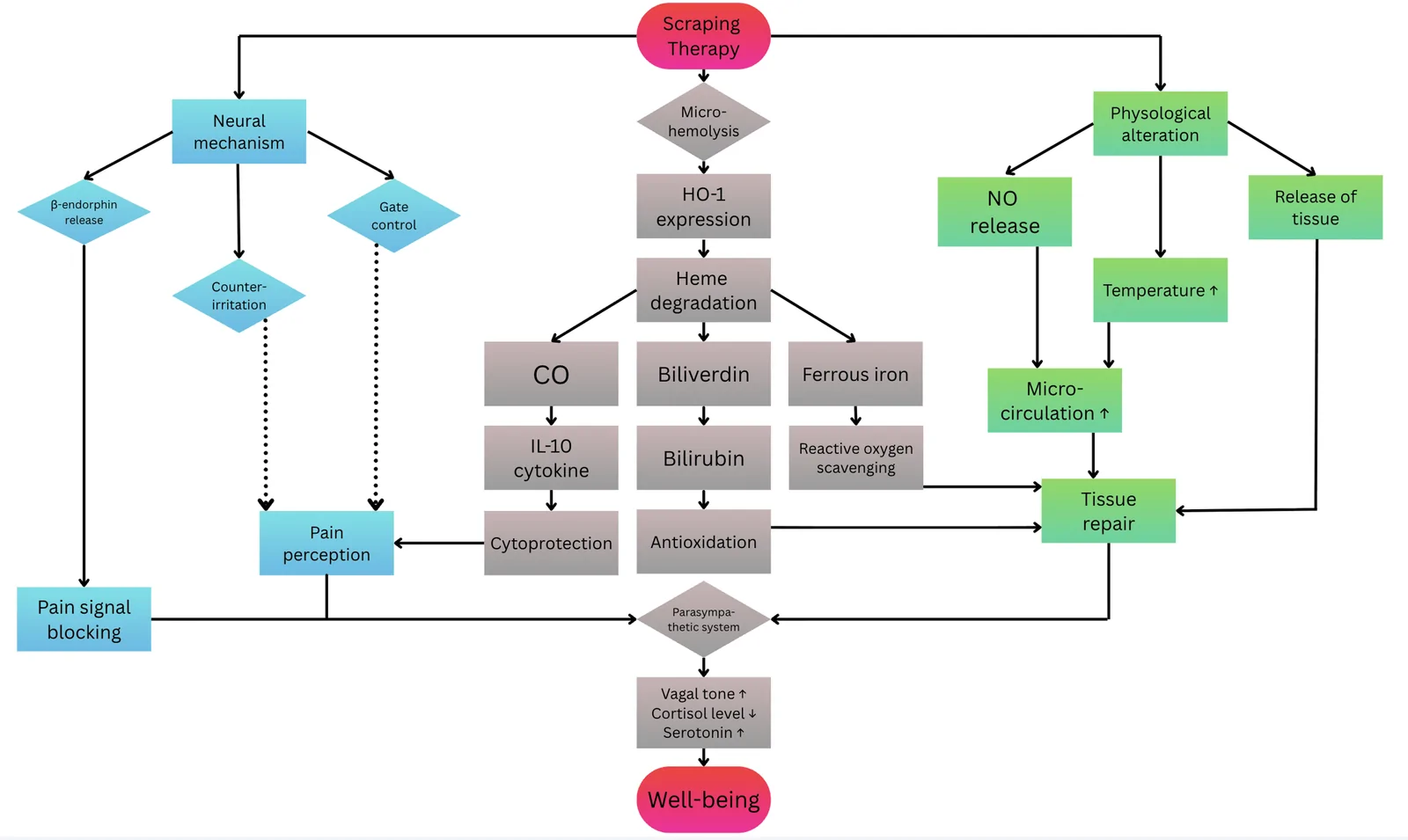
Proposed mechanisms underlying scraping therapy Scraping breaks capillaries and releases erythrocytes into the subcutis. This boosts HO-1 and leads to microhemolysis. The breakdown produces substances like CO, biliverdin, and Fe²⁺, exerting systemic anti-inflammatory and antioxidant effects. These changes help retrain the immune system, balancing cytokines to lower inflammation. Pain eases through pain gate control, counter-irritation, and the release of β-endorphins and NO. Mood improves due to stronger parasympathetic activity, more vagal tone, less cortisol, and higher serotonin. Solid arrows mean activation; dashed lines mean suppression. HO-1: heme oxygenase-1; CO: carbon monoxide; NO: nitric oxide

Cortisol is a primary stress hormone linked to stress, anxiety, and depression. Massage therapy may reduce cortisol levels by promoting parasympathetic activity [27]. In a randomized controlled trial focusing on perimenopausal women, those undergoing weekly scraping treatments demonstrated marked improvements in fatigue, sleep difficulties, and depressive symptoms relative to the control group [28]. Clinical research of 56 Parkinson's patients revealed that scraping therapy alleviated symptoms and enhanced sleep quality while also boosting levels of serotonin and IL-10 and reducing IL-8 [8]. Since serotonin plays a key role in mood regulation, pain perception, and sleep patterns, the rise in serotonin levels following scraping therapy likely accounts for the psychological benefits reported by participants [27]. The self-constructed flowchart shows a clear picture of the whole therapeutic mechanism (Figure 1).

Discussion

Rather than suggesting Gua Sha "works" because of microhemolysis, it is more appropriate to say microhemolysis provides a straightforward and empirically grounded mechanism for its therapeutic effects. According to this theory, petechiae are not coincidental but rather the main cause of the therapy's benefits, offering a model that is both testable and biologically consistent.

The following components are each supported by at least one primary study indexed in PubMed: alterations in mechanical microperfusion [2,10], systemic induction of HO-1 [1,14], suppression of pro-inflammatory cytokines [11,21], increased immune response [5,8], elevated tissue β-endorphin [21,22], autonomic recalibration [23], and clinical pain relief [3,8,17]. By construction, it offers a systemic perspective that integrates the immune, nervous, and endocrine systems into a unified model. 

While current literature demonstrates a plausible link connecting mechanical microhemolysis to HO-1 expression, systemic cytokine modulation, and pain attenuation, it is critical to distinguish between established biological correlations and proven causal mechanisms. Much of the direct tissue biomarker data, such as localized HO-1 upregulation, heme oxygenase pathway activation, and specific spinal neurochemical changes, is derived from preclinical rodent models [4,21]. In contrast, human trials primarily measure clinical endpoints such as pain scores, range of motion, and surface microcirculation [5,23]. Consequently, while rodent studies demonstrate physiological responses that parallel human clinical improvement, a definitive cause-and-effect relationship in humans remains unproven.

Even with a clear understanding of how it works, there remain major limits. Most clinical studies have been carried out on rats [1,5-7,11,18,21], meaning that robust and carefully designed clinical trials are necessary before these results can be reliably translated to human applications. Moreover, the heterogeneity across current controlled trials, varying in methodologies [1-3,5-8,11,17,18,21-23,27,28], instruments [1,6,10,17,18], treatment periods [1,2,5-8,10,11,17,18,21,22,27,28], pressure levels [3,6,10], and methods of measuring results [1-3,5-8,10,11,17,18,21-23,27,28], hinders direct comparisons and limits the ability to pool data for robust meta-analyses. Currently, there is no convincing evidence of cause-and-effect linkages between these processes. To find that proof, scientists need specialized investigations such as receptor blocking, HO-1 inhibition, and dose-response analyses. Furthermore, to ensure reproducibility across future translational human and preclinical animal models, researchers must prioritize the standardization of biomechanical application parameters. Specifically, future protocols should rigorously measure and control scraping force/pressure (quantified in Newtons), instrument application angle, stroke frequency, and treatment duration. Standardizing these physical inputs will be essential for establishing precise dose-dependent physiological relationships and comparing outcomes across biomedical studies.

To transition this framework from a plausible model to a proven medical therapy, future interventional trials must specifically attempt to prove, or disprove, the necessity of the microhemolytic cascade. First, translational human trials should incorporate specific biomarker assays (e.g., serial serum HO-1, CO, and plasma β-endorphin levels) paired alongside validated clinical outcome measures to determine if biomarker magnitude directly correlates with therapeutic efficacy. Second, prospective clinical studies should compare standard petechiae-inducing scraping against a biomechanically matched, non-petechial sham intervention (e.g., light-touch mobilization) to isolate the therapeutic role of extravasated blood breakdown. Third, preclinical models must utilize targeted molecular interventions, such as HO-1 gene knockouts, pharmacological HO-1 inhibitors (e.g., zinc protoporphyrin), or specific opioid receptor antagonists (e.g., naloxone), to test whether blocking the microhemolytic-HO-1 pathway completely abolishes the observed analgesic and anti-inflammatory effects.

## Conclusions

In summary, scraping therapy represents a promising non-pharmacological intervention whose biological effects extend beyond simple localized tissue friction. Importantly, current evidence suggests a single initiating process, microhemolysis, that mechanistically links the intentionally induced petechial markings directly to subsequent downstream anti-inflammatory and analgesic benefits, as opposed to viewing petechiae as a mere epiphenomenon. By framing extravasated erythrocyte breakdown and subsequent HO-1 induction as the primary biological driver, this model provides a falsifiable hypothesis. Rigorous interventional trials specifically designed to test, validate, or disprove this microhemolytic cascade should now be conducted.
